# Crocins ameliorate acute kidney injury in glycerol-induced rhabdomyolysis by targeting the PLIN1/PPARs signaling pathway

**DOI:** 10.3389/fphar.2026.1874817

**Published:** 2026-09-11

**Authors:** Fangbo Zhang, Yu Li, Qingqing Cai, Ye Zhao, He Xu, Huamin Zhang, Hongjun Yang

**Affiliations:** 1 Institute of Chinese Materia Medica, China Academy of Chinese Medical Sciences, Beijing, China; 2 Institute of Basic Theory of Chinese Medicine, China Academy of Chinese Medical Sciences, Beijing, China; 3 China Academy of Chinese Medical Sciences, Beijing, China

**Keywords:** acute kidney injury, crocins, rhabdomyolysis, network analysis, PLIN1, PPARs

## Abstract

**Background:**

Rhabdomyolysis (RM) is a potentially life-threatening syndrome characterized by skeletal muscle damage, with acute kidney injury (AKI) being its most severe complication. Currently, no effective treatment exists for RM-induced AKI. Crocins, the major bioactive constituents extracted from the stigma of *Crocus sativus* L. (Saffron), possess diverse pharmacological activities.

**Objectives:**

This study aimed to explore the pharmacological effect and possible mechanism of crocins in the treatment of RM-induced AKI.

**Method:**

Network analysis was first applied to predict potential targets of crocins against RM. A rat model of hypertonic glycerol-induced RM was then established to evaluate pharmacological effect, including assessment of muscle and renal pathology, inflammatory cytokines, biochemical markers, and oxidative stress-related enzymes. Tandem mass tag–based quantitative proteomics was further employed to identify key disease targets. Molecular docking was then conducted to validate potential target interactions of crocins in RM-induced AKI treatment.

**Results:**

Animal experiments demonstrated that crocins alleviated muscle and renal injuries by inhibiting inflammation and oxidative stress while preserving hepatic and renal functions. Proteomic analysis identified perilipin 1 (PLIN1) as a critical candidate biomarker mediating these effects. Both network analysis and proteomics indicated that the peroxisome proliferator-activated receptors (PPARs) signaling pathway was closely involved in protective mechanism. Furthermore, Western blotting confirmed that crocins exerted pharmacological effect through regulating the PLIN1/PPARs signaling pathway. Molecular docking revealed that the best docking activities were demonstrated by crocin I/II–PLIN1, crocin I–PPARα, and crocin II–PPARγ.

**Conclusion:**

Crocins mitigated RM-induced AKI primarily by suppressing inflammation and oxidative stress via the PLIN1/PPARs signaling pathway. These findings provide a scientific and theoretical basis for the potential clinical application of crocins in treating RM-induced AKI.

## 1 Introduction

Rhabdomyolysis (RM) is a frequent clinical syndrome characterized by acute skeletal muscle injury, leading to the release of intracellular components, involving potassium, phosphate, urate, myoglobin, creatine kinase, aldolase, and aspartate aminotransferase, into the circulation and extracellular fluid. The main clinical manifestations are myalgia, weakness, and dark “tea-colored” urine. When excessive myoglobin surpasses the binding capacity of plasma globulins, it is filtered through the glomeruli into renal tubules, where it contributes to tubular obstruction and subsequent renal dysfunction (Cabral et al., 2020; Gupta et al., 2021). The most severe complication is acute kidney injury (AKI), driven by massive myoglobinuria, which develops in approximately 10%–50% of patients with RM (Legrand et al., 2024). RM can arise from a wide range of causes, including extreme physical exertion, alcohol use, toxins, drugs, infections, trauma, electrical shock, crush injuries, exposure to extreme temperatures, and both hereditary and acquired metabolic myopathies (Richert et al., 2026). Prognosis of RM is largely determined by the underlying etiology and management of complications, particularly AKI (Morin et al., 2024). Current treatment primarily focuses on preserving renal function through supportive care, prevention and management of AKI, blood purification strategies, and renal replacement therapy in severe cases (Graf et al., 2024; Subashri et al., 2023). However, no effective targeted therapy for RM-induced AKI is available, highlighting urgent need for novel preventive and therapeutic approaches.

Traditional Chinese medicine (TCM) has been widely used to prevent and treat renal diseases for centuries. Accumulating evidence shows that phytochemicals derived from TCM herbs contribute to their renoprotection against various AKI models (Wang et al., 2025; Chen et al., 2025). Astragalus polysaccharide potentially mitigated RM-induced AKI by impeding the M1 macrophage polarization and the cGAS-STING pathway (Sun et al., 2024). Triterpenoid components in *Poria cocos* and its surface layer exert the renoprotective effect through improving AKI and protecting against renal fibrosis (Guo et al., 2025). A retrospective study confirms that a combination of *Radix Astragali* and *Salvia miltiorrhiza* Bunge increased the rate of short-term recovery from AKI and potentially prevented progression to chronic kidney disease (He et al., 2025). Numerous studies have suggested that quercetin can effectively attenuate AKI by inhibiting renal inflammation, ferroptosis, and cell apoptosis (Wang and Wu, 2025). Despite promising preclinical evidence, these findings remain largely experimental, and rigorous clinical trials are urgently needed to validate their efficacy and safety in patients (Zhou et al., 2026).

Crocins (CRCs) are the primary bioactive constituents extracted from the stigma of *Crocus sativus* L. (saffron). They are mainly hydrophilic carotenoids, including crocins, picrocrocin, and safranal (Kakouri et al., 2020). CRCs exhibit a broad spectrum of pharmacological activities, such as antioxidant, antitumor, anti-inflammatory, antidepressant, antidiabetic, hepatoprotective, and nephroprotective effects (Hua et al., 2024). Research has shown that crocin alleviated membranous nephropathy by inhibiting immune injury and podocyte damage through activating the Sirt1/Nrf2/HO-1 pathways (Liu et al., 2023).

Safranal ameliorated renal damage, inflammation, and podocyte injury in membranous nephropathy via the SIRT/NF-κB signaling (Bao et al., 2025). Saffron and its constituents significantly prevented biochemical and histopathological changes, and thus displayed nephroprotective effects mediating via antioxidation, anti-apoptosis, and anti-inflammation (Zarei and Elyasi, 2022). In this study, we evaluated the effects of CRCs on glycerol-induced RM and AKI in rats and then explored its potential mechanism. This research offers a new strategy for the prevention and treatment of RM-associated AKI. Briefly, network analysis was applied to predict the potential mechanism of CRCs against RM, providing a theoretical basis for mechanistic exploration. A hypertonic glycerol-induced RM rat model was then established to validate molecular targets identified through network analysis. Furthermore, quantitative proteomics and Western blotting were employed to uncover therapeutic targets involved. Collectively, our study demonstrated that CRCs relieved RM-induced AKI primarily by suppressing inflammation and oxidative stress via the PLIN1/PPARs signaling pathway. Our findings provide evidences of both pharmacological effect and underlying mechanism of CRCs in protecting against RM-induced AKI.

## 2 Materials and methods

### 2.1 Experimental animals and drugs

Male SPF Sprague-Dawley rats (200–240 g) were purchased from Beijing Vital River Laboratory Animal Technology Co., Ltd., China (license No. SCXK (Jing) 2019-0003). All animal experiments were conducted in accordance with ethical guidelines for animal care and use approved by the Animal Research Committee of the Institute of Chinese Materia Medica, China Academy of Chinese Medical Sciences (Approval No. 2020B101). CRCs, the primary constituents of the Saffron Total Glycoside Tablet (*Xihonghua Zonggan Pian*), were supplied by Reyoung Pharmaceutical Co., Ltd., China. The positive control drug, catechin (powder, purity ≥98%), was obtained from Beijing Solarbio Science and Technology Co., Ltd., China (Chander et al., 2003). Clinical oral dose of the Saffron Total Glycoside Tablet is 2.4 mg/kg/day for a 60 kg adult (12 mg/tablet, 4 tablets per dose, 3 times daily), equivalent to a rat (200 g) gavage dose of 14.88 mg/kg/day. Based on this calculation, gavage doses of 7.5, 15.0, and 30.0 mg/kg/day were selected for animal experiment.

### 2.2 Chemical analysis of CRCs by UPLC method

CRCs samples were analyzed using a Waters XEVO TQ-S Micro mass spectrometer coupled with a Waters Acquity I-Class UPLC system. Separation was performed on a Waters UPLC HSS T3 column (1.8 μm, 2.1 mm × 100 mm). The mobile phase consisted of 0.1% formic acid in water (A) and acetonitrile: isopropanol (1: 9) with 5 mM ammonium acetate and 0.1% formic acid (B). The flow rate was set at 0.3 mL/min, and the column temperature was maintained at 45 °C. The optimized gradient program was: 0–1.5 min, 10% B; 1.5–6 min, 10%–85% B; 6–7 min, 85%–97% B; 7.5–7.6 min, 97%–10% B; and 7.6–9.0 min, 10% B. The injection volume was 3.0 µL.

### 2.3 Network analysis of CRCs in treating RM

Chemical constituents of CRCs were mainly identified through literature retrieval. Molecular targets of these compounds were obtained from the BATMAN-TCM database (http://bionet.ncpsb.org/batman-tcm/). RM-related disease targets were collected from the OMIM (https://omim.org/) and GeneCards (https://www.genecards.org/) databases. The potential targets of CRCs and RM-associated disease targets were then integrated and imported into the STRING database (http://www.string-db.org/) to construct a protein–protein interaction (PPI) network. The PPI network was visualized using the Cytoscape software (version 3.7.2, Cytoscape Consortium, Boston, MA, United States). Functional enrichment, including Gene Ontology (GO) and Kyoto Encyclopedia of Genes and Genomes (KEGG) pathway analysis, was conducted via the DAVID database (https://david.ncifcrf.gov/).

### 2.4 Animal model construction and group design

The rat model of RM was established as previously described (Al-Kharashi et al., 2023). Briefly, rats were anesthetized via intraperitoneal injection of pentobarbital sodium (40 mg/kg, Cat. P3761, Sigma, MA, United States) and then given a single intramuscular injection of 50% glycerol (10.0 mL/kg in sterile saline; Solarbio, Beijing, China) into the outer right hind limb muscle. After 3 days of acclimatization, rats were randomly assigned to six groups (n = 12 each): control, RM model, CRCs low-dose (7.5 mg/kg), CRCs medium-dose (15.0 mg/kg), CRCs high-dose (30.0 mg/kg), and catechin (2.0 mg/kg). CRCs and catechin were administered once daily by gavage for seven consecutive days, followed by model induction on day 8. Model group rats received distilled water for 7 days before glycerol injection, while control rats received distilled water and then an equal volume of saline injection. All rats were intraperitoneally anesthetized with pentobarbital sodium (40 mg/kg, Cat. P3761, version 3.7.2, Sigma, Boston, MA, United States) at 24 h after glycerol injection. Blood samples were collected via abdominal aorta. The injected muscle and one kidney were fixed in 10% formalin (Cat. P1110, Solarbio, Beijing, China), whereas the contralateral kidney was rinsed with ice-cold saline and snap-frozen in liquid nitrogen.

### 2.5 Histopathological examination

Muscle and kidney tissues were embedded in paraffin, sectioned into 5 μm slices, and stained with hematoxylin-eosin (HE). Additionally, muscle samples were subjected to Mallory’s phosphotungstic acid-hematoxylin (PTAH) staining. Histological changes were examined under a light microscope (Olympus, Tokyo, Japan), and five random fields were photographed from each slide. Tissue injury was scored as follows: 0, no tubular or myofiber injury; 1, <10% injury; 2, 10%–25% injury; 3, 26%–50% injury; 4, 51%–75% injury; and 5, >75% injury (Kurus et al., 2009). The degree of tissue injury was detected blindly by two independent investigators in a blinded manner.

### 2.6 Detection of serum biomarkers and inflammatory cytokines

Blood samples were centrifuged at 1,500 rpm for 10 min to obtain serum. Hepatic function markers (alanine aminotransferase, ALT, Cat. C009-2-1; aspartate aminotransferase, AST, Cat. C010-2-1), renal function markers (blood urea nitrogen, BUN, Cat. C013-3-1; serum creatinine, Scr, Cat. C011-2-1; creatine kinase, CK, Cat. A032-2-1), and oxidative stress-related enzymes (lactate dehydrogenase, LDH, Cat. A020-2-2; superoxide dismutase, SOD, Cat. A001-3-2; malondialdehyde, MDA, Cat. A001-3-2) were all measured using commercial biochemical kits (Nanjing Jiancheng, Jiangsu, China). Serum levels of inflammatory cytokines, including interleukin (IL)-1β (Cat. CSB-E08055r), IL-6 (Cat. CSB-E04640r), tumor necrosis factor (TNF)-α (Cat. CSB-E11987r), transforming growth factor (TGF)-β1 (Cat. No.), and monocyte chemoattractant protein (MCP)-1 (Cat. CSB-E07429r), were determined with ELISA kits (Wuhan Cusabio, Hubei, China) following the manufacturer’s instructions.

### 2.7 Serum and renal myoglobin measurement

Serum myoglobin (Mb) was measured using an ELISA kit (Cat. H150-1-2, Nanjing Jiancheng, Jiangsu, China) following the manufacturer’s instruction. For renal tissue detection, an equal weight of kidney tissue was homogenized in saline at a ratio of 1 g: 5 mL. The homogenate was sonicated (Ningbo Xinzhi, Zhejiang, China) and centrifuged at 12,000 g for 15 min. The protein concentration of the supernatant was determined using a bicinchoninic acid (BCA) protein assay kit (Cat.23227, Thermo Fisher Scientific, MA, United States). Mb level in renal tissue was then quantified with a biochemical kit (Cat. H150-1-1, Nanjing Jiancheng, Jiangsu, China), according to the manufacturer’s instruction.

### 2.8 Immunohistochemical staining and scoring

Paraffin-embedded renal tissue sections (5 μm) were deparaffinized, rehydrated in graded alcohol, and treated with 3% hydrogen peroxide in methanol for 5 min to block endogenous peroxidase activity. Antigen retrieval was performed in citrate buffer using microwave heating for 8 min. Sections were incubated overnight at 4 °C with rabbit monoclonal anti-Mb antibody (1:300, Cat. ab77232, Abcam, MA, United States). After sequential incubation with a biotinylated secondary antibody and streptavidin–alkaline phosphatase, immunoreactivity was visualized with DAB substrate (Cat. K5007, Dako, Copenhagen, Denmark). Renal Mb expression was evaluated semi-quantitatively using the Remmele immunoreactive score (Remmele and Stegner, 1987). The proportion of positively stained tubular epithelial cells was graded on a 0–4 scale: 0 = 0%, 1 = <10%, 2 = 10%–50%, 3 = 51%–80%, 4 = >80%. All scoring was performed in a blinded manner, and mean scores were calculated per rat and per group.

### 2.9 Tandem mass tag (TMT)-based quantitative proteomics

Renal tissues from control, model, and CRCs 30.0 mg/kg groups (n = 5/group) were analyzed. Samples were lysed in RIPA buffer (Cat. R0010, Solarbio, Beijing, China) mixed with protease inhibitors (Cat. P2714, Sigma, MO, United States), centrifuged at 13,000 g for 15 min at 4 °C, and protein concentrations were determined using a BCA kit (Cat.23227, Thermo Fisher Scientific, MA, United States). Proteins were digested with trypsin and labeled with 16-plex TMT reagents (Cat. A44521, ThermoFisher Scientific, MA, United States) according to the manufacturer’s instructions. Labeled peptides were fractionated by an Agilent 1100 HPLC system, vacuum dried, dissolved in 0.1% formic acid, and analyzed on an EASY-nLC1200 coupled to an Orbitrap Elite mass spectrometer (ThermoFisher Scientific, CA, United States). Functional enrichment analysis (GO and KEGG) was conducted using the DAVID database (https://david.ncifcrf.gov/). Differentially expressed proteins (DEPs) were identified with thresholds of *p* < 0.05, fold change ≥1.5 (upregulated), or ≤0.7 (downregulated) (Ba et al., 2022).

### 2.10 Western blotting

Renal tissues were lysed and sonicated in ice-cold RIPA buffer with protease inhibitors (Cat. P6730, Solarbio, Beijing, China). Lysates were centrifuged at 13,000 g for 15 min at 4 °C, and supernatants were collected. Protein concentrations were determined using a BCA kit (Cat.23227, Thermo Fisher Scientific, MA, United States). Equal amounts of protein were separated by SDS-PAGE and transferred to PVDF membranes (Cat. IPFL00010, Millipore, Darmstadt, Germany). Membranes were incubated overnight at 4 °C with primary antibodies against Perilipin 1 (1:1,000, Cat. ab3526, Abcam, MA, United States), Peroxisome proliferator-activated receptor (PPAR)α (1:1,000, Cat. ab227074, Abcam, MA, United States), PPARγ (1:1,000, Cat. sc-81152, Santa Cruz Biotechnology, CA, United States), and GAPDH (1:1,000, Cat. 5174, Cell Signaling Technology, MA, United States) as an internal control. After incubation with secondary antibodies, protein bands were detected using an enhanced chemiluminescence reagent (Cat. WBKLS0100, Millipore, MA, United States).

### 2.11 Molecular docking

The three-dimensional (3D) structures of the compounds were retrieved from the PubChem database (http://pubchem.ncbi.nlm.nih.gov/). The 3D crystal structures of the three molecular targets PLIN1, PPARα, and PPARγ were downloaded from the Worldwide Protein Data Bank database (https://www.rcsb.org/). Before molecular docking, the crystal structure was preprocessed using the PyMol 3.1.3, including protonation, assignment of partial charges, addition of hydrogens, removal of crystal water molecules, and energy minimization-based structural optimization. Molecular docking of the receptor and ligand as well as free-binding energies were performed using the online CB-Dock2 platform (https://cadd.labshare.cn/cb-dock2/php/index.php).

### 2.12 Statistical analysis

Data were analyzed using Statistical Package for the Social Sciences version 20.0 (IBM, Armonk, NY, United States). Results are expressed as mean ± standard deviation (SD). Comparisons among groups were made using Student’s *t*-test or one-way analysis of variance. A *p*-value < 0.05 was considered statistically significant. The normality of data distribution for quantitative analysis was verified using the Shapiro–Wilk test before parametric statistical tests.

## 3 Results

### 3.1 Content determination of CRCs sample

**FIGURE 1:**
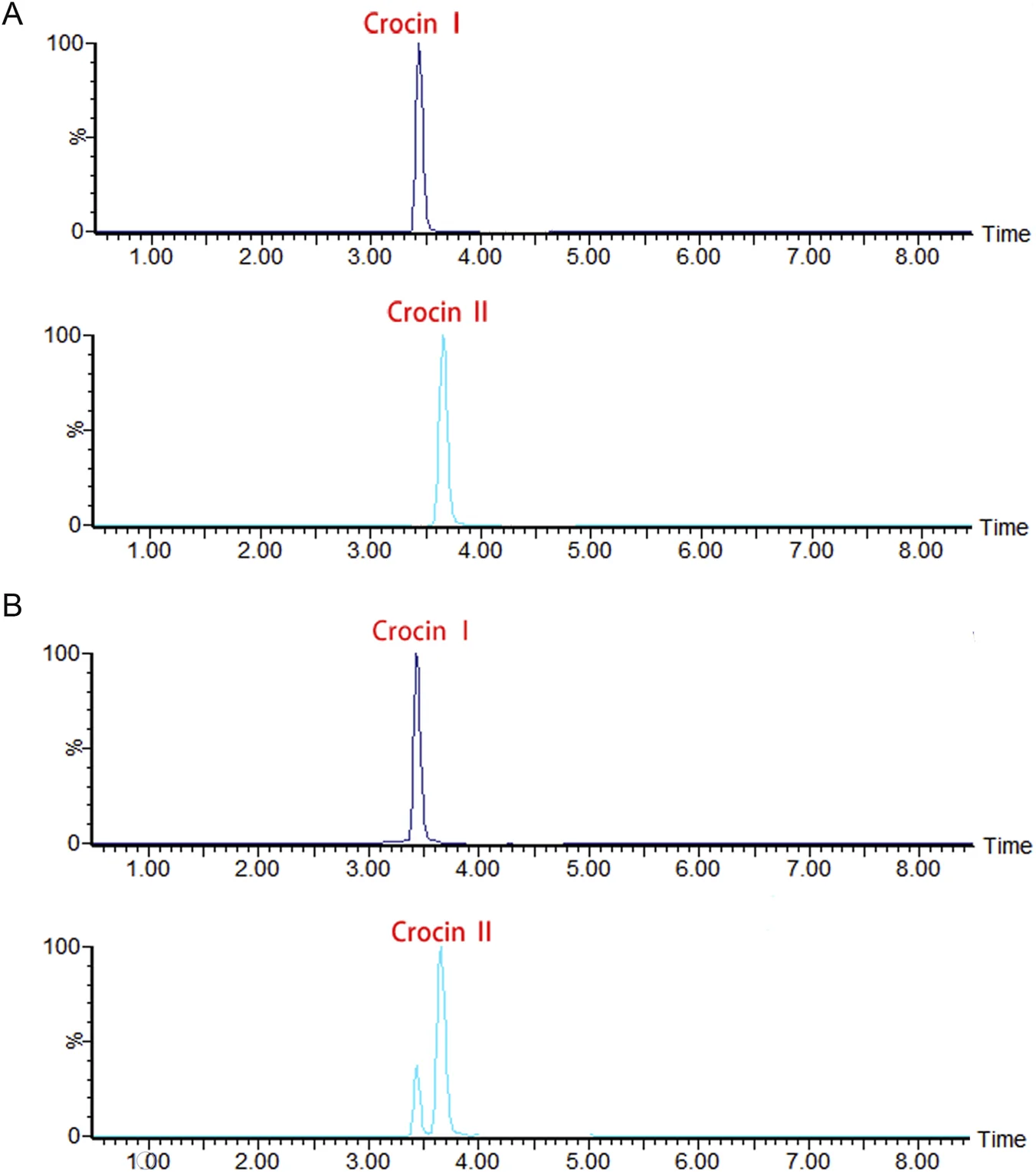
Extracted ion chromatograms of the reference standards **(A)** and the CRCs samples **(B)**, with crocin I and crocin II labeled in the image.

The primary quantified components of CRCs samples were crocin I and crocin II, with contents of 52.32 and 20.63 mg/g, respectively. Extracted ion chromatograms of the reference standards and CRCs samples are shown in Figure 1.

### 3.2 Network analysis predicted potential mechanism of CRCs against RM

**FIGURE 2:**
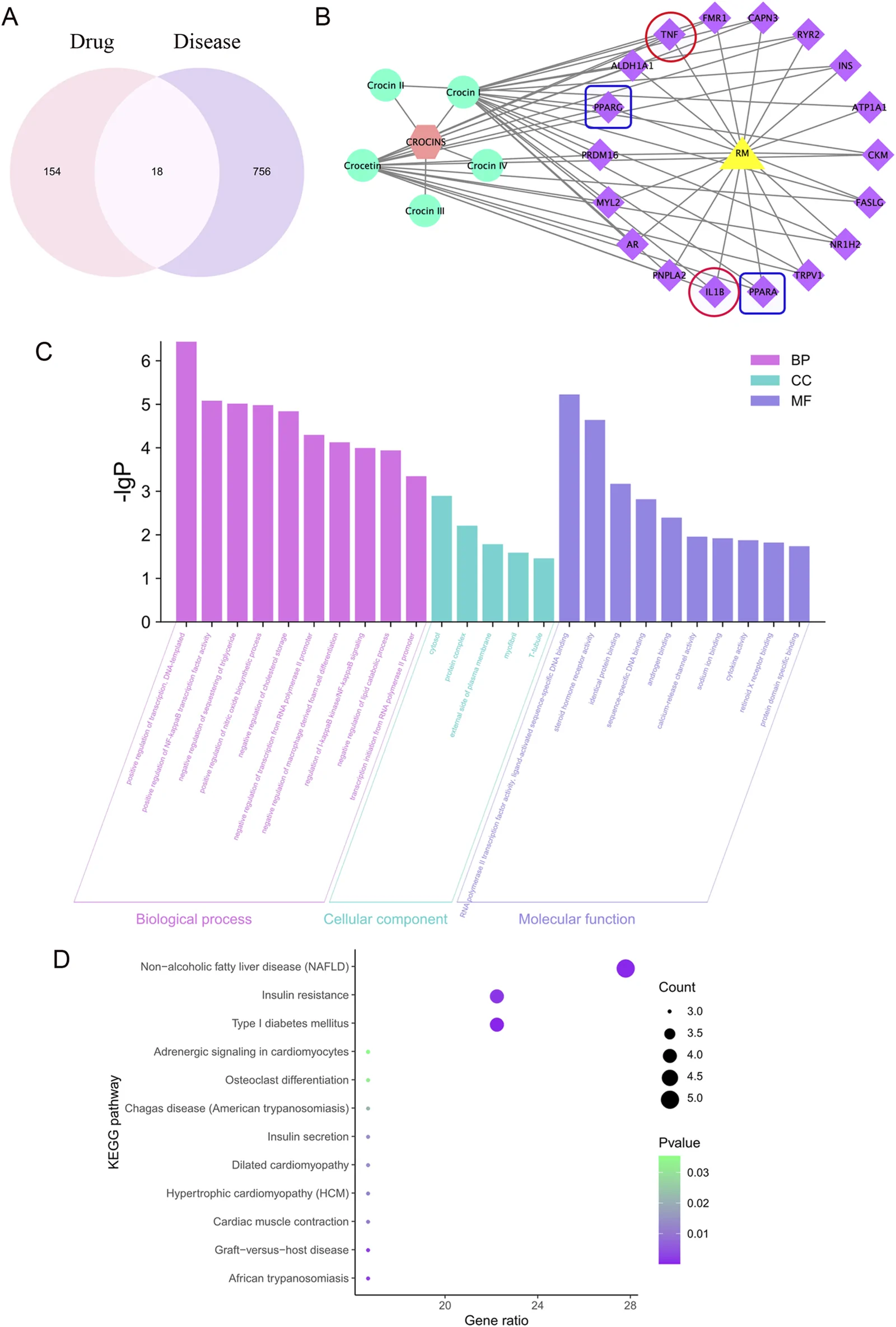
Network analysis of CRCs for RM treatment. **(A)** Venn diagram depicting 18 common genes shared between CRCs targets and RM targets. **(B)** Drug–component–target interaction network, with CRCs active compounds shown in green and the 18 overlapping target genes in purple. **(C)** GO functional analysis highlighting the top 10 significantly enriched terms in BP, CC, and MF. **(D)** KEGG pathway analysis illustrating the top 12 enriched signaling pathways.

The main constituents of CRCs include crocin I, crocin II, crocin III, crocin IV, and crocetin, as reported in the literature (Song et al., 2021). A total of 172 drug-related targets of CRCs and 774 molecular targets of RM were collected, among which 18 overlapping genes were identified (Figure 2A). These shared targets were submitted to the STRING database to build a protein–protein interaction (PPI) network. As shown in Figure 2B, two inflammatory cytokines (TNF and IL-1β) and signaling molecules (PPARα and PPARγ) occupied central positions in the PPI network, suggesting that they may play crucial roles in the pathogenesis of RM and pharmacological effect of CRCs.

GO enrichment analysis revealed that biological process (BP) terms were mainly associated with positive regulation of DNA-templated transcription, activation of NF-κB transcription factor activity, inhibition of triglyceride sequestration, stimulation of nitric oxide biosynthesis, and suppression of cholesterol storage. Cellular component (CC) terms included cytosol, protein complex, external plasma membrane, myofibril, and T-tubule. Molecular function (MF) terms were related to RNA polymerase II transcription factor activity, ligand-activated sequence-specific DNA binding, steroid hormone receptor activity, identical protein binding, and androgen binding (Figure 2C). KEGG enrichment analysis further indicated that the main signaling pathways were involved in type I diabetes mellitus, non-alcoholic fatty liver disease, insulin resistance, cardiac muscle contraction, and hypertrophic cardiomyopathy (Figure 2D).

### 3.3 CRCs relieved muscular and renal injuries in RM-induced AKI rats

**FIGURE 3:**
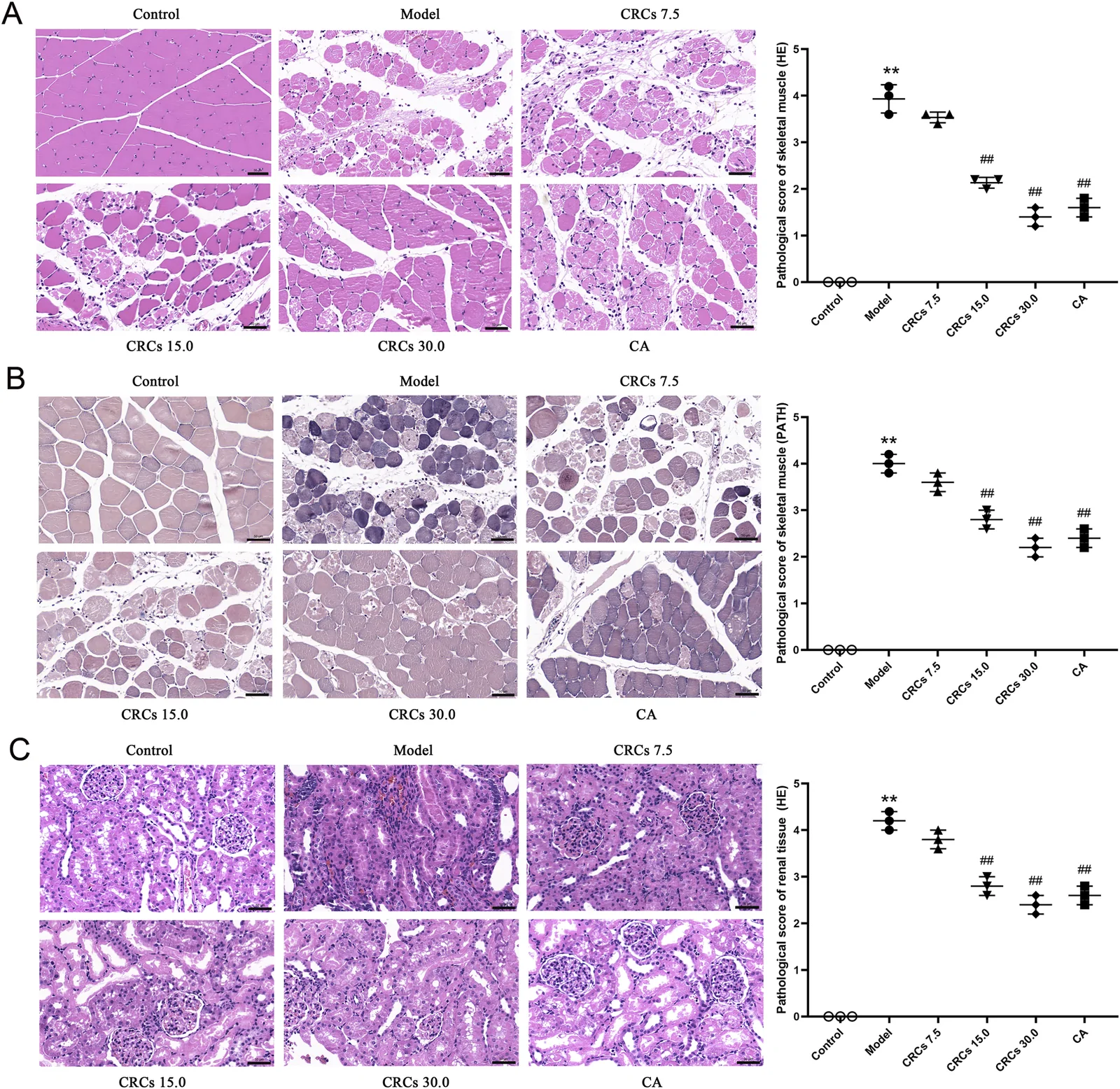
CRCs alleviated muscular and renal damages in RM-induced AKI rats. **(A)** Histopathological changes in skeletal muscle assessed by HE staining. Scale bar = 50 μm, n = 3. **(B)** Evaluation of myofiber injury using PTAH staining. Scale bar = 50 μm, n = 3. **(C)** Histopathological changes in renal tissue observed by HE staining. Scale bar = 50 μm, n = 3. Data are presented as mean ± SD. **p < 0.01 vs. control group; ^#^ *p* < 0.05, ^##^ *p* < 0.01 vs. model group.

HE staining of skeletal muscle at the injection site showed that muscle fibers in the control group were intact and well organized (Figure 3A). In contrast, the model group exhibited reduced fiber density, disrupted alignment, swollen fibers, loss of striations, vacuolization, and extensive inflammatory cell infiltration. CRCs treatment improved muscle structure, with the high-dose group showing the most pronounced recovery. PTAH staining further revealed normal morphology and color in the control group (Figure 3B). The model group displayed extensive purple-blue or light purple-blue staining, irregular fiber arrangement, increased gaps, and a honeycomb-like appearance. These pathological changes were visibly ameliorated by CRCs treatment. Histopathological scores from HE and PTAH staining confirmed that CRCs significantly reduced skeletal muscle injury in RM-induced AKI rats.

HE staining of renal tissue indicated normal glomerular and tubular structures in the control group (Figure 3C). However, the model group showed that the glomeruli were congestive and swollen with balloon dilatation. The proximal renal tubular epithelial cells were swollen, and the tubes were dilated and arranged disorderedly. The capillaries were congested with many red blood cells. A large amount of inflammatory cells was penetrated into the extracellular interstitium. Myoglobin casts in some glomeruli were formed. The CRCs-treated groups showed a reduction in congestion and swelling of the glomeruli and tubules, a decrease in myoglobin cast and capillary congestion, and a decline in inflammatory cells infiltration, thus visibly alleviating renal injury. The high-dose CRCs group exhibited the greatest structural recovery. Histopathological scoring further validated the protective effect of CRCs against RM-induced AKI.

**FIGURE 4:**
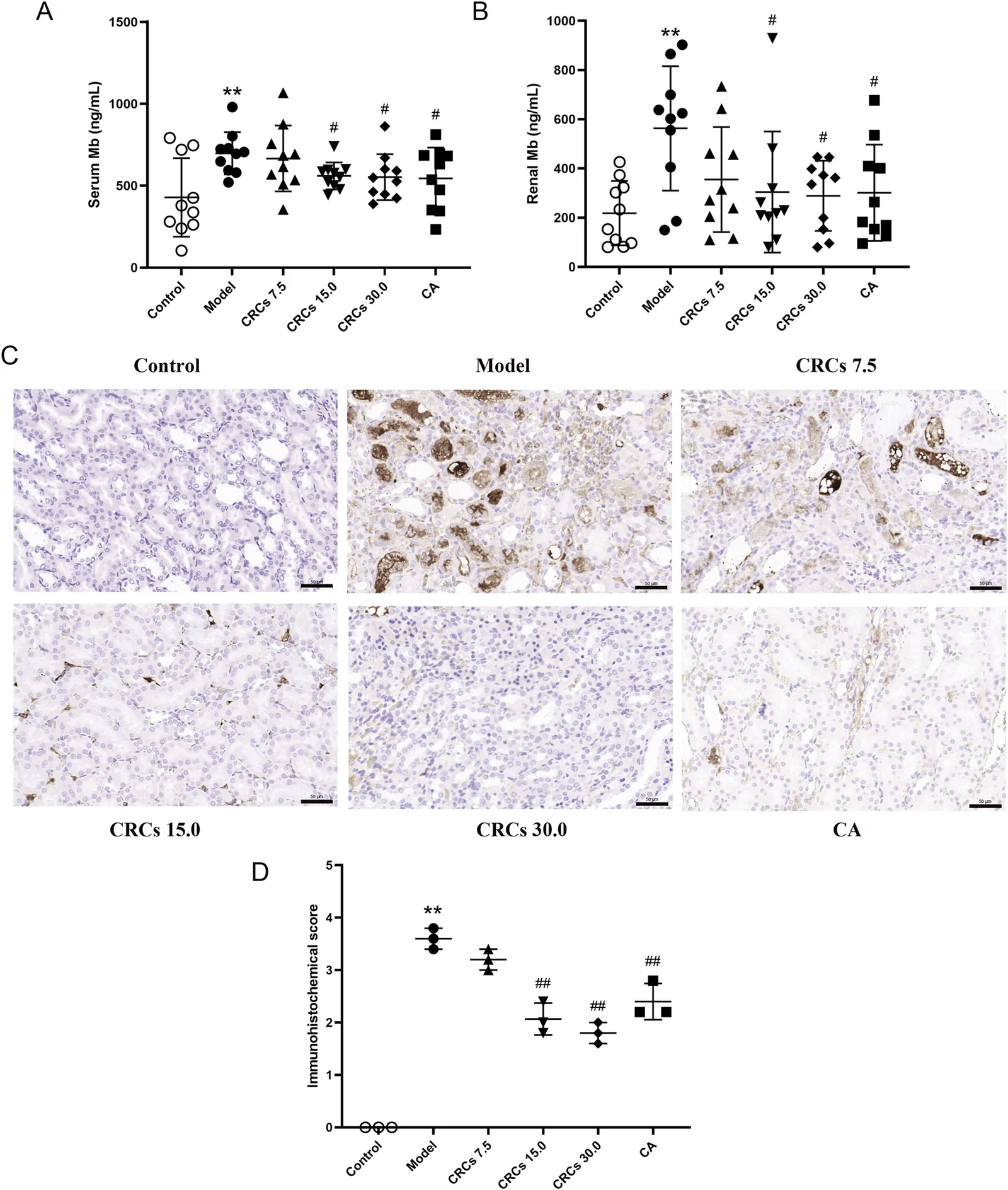
CRCs reduced Mb levels in serum and renal tissues of RM-induced AKI rats. **(A)** Serum Mb level determined by ELISA, n = 10. **(B)** Renal Mb level measured using a biochemical kit, n = 10. **(C)** Representative immunohistochemical images of renal Mb. Scale bar = 50 µm. **(D)** Semi-quantitative immunohistochemical scoring of renal Mb, n = 3. Data are presented as mean ± SD. **p < 0.01 vs. control group; ^#^ *p* < 0.05, ^##^ *p* < 0.01 vs. model group.

Biochemical analysis showed that serum and renal Mb levels were significantly elevated in the model group compared with the control group (Figures 4A,B). Both medium- and high-dose CRCs groups exhibited marked reductions in Mb levels. Immunohistochemical staining revealed no detectable Mb in the control group (Figure 4C), whereas the model group showed strong positive staining, with Mb distributed in glomeruli, proximal tubules, and distal tubules. Brown to dark-brown staining indicated positive and strongly positive expression, respectively. Immunohistochemical scoring confirmed that Mb expression progressively declined in CRCs-treated groups, with the lowest levels observed in the high-dose group (Figure 4D). These results demonstrated that CRCs mitigated muscle and kidney injuries in RM-induced AKI rats by lowering serum and renal Mb levels.

### 3.4 CRCs protected hepatic and renal functions in RM-induced AKI rats

**FIGURE 5:**
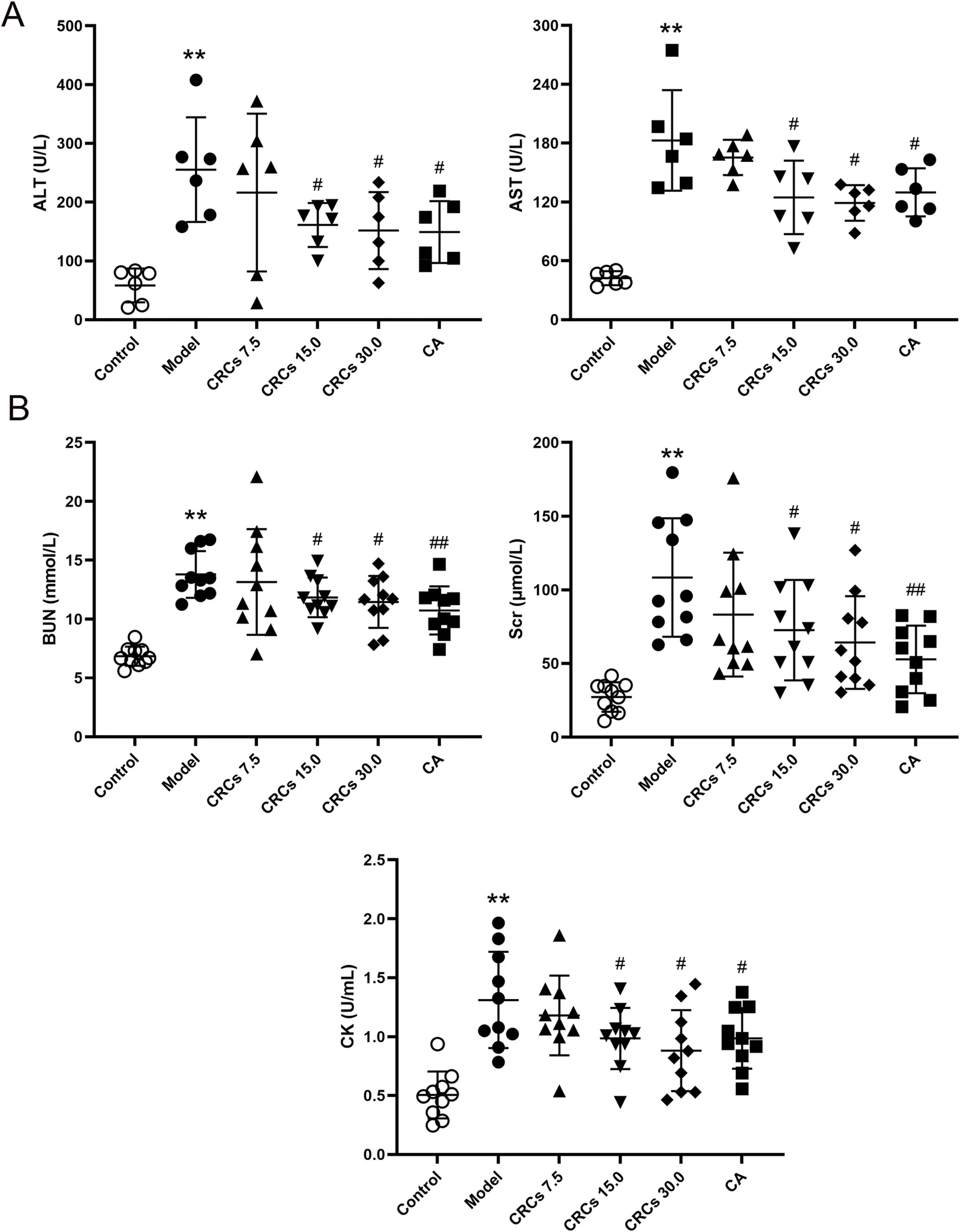
CRCs preserved hepatic and renal functions in RM-induced AKI rats. **(A)** Serum hepatic function markers, ALT and AST, measured by biochemical kits, n = 6. **(B)** Serum renal function markers, including BUN, Scr, and CK, assessed with biochemical kits, n = 10. Data are presented as mean ± SD. **p < 0.01 vs. control group; ^#^ *p* < 0.05, ^##^ *p* < 0.01 vs. model group.

In comparison with the control group, the levels of liver function markers ALT and AST were significantly elevated in the model group (Figure 5A). CRCs treatment at medium and high doses led to a marked reduction of both ALT and AST. Similarly, renal function indicators, including BUN, Scr, and CK, were markedly increased in the model group compared with the control group (Figure 5B). These elevations were significantly reduced in rats treated with CRCs at doses of 15.0 and 30.0 mg/kg. Collectively, these findings suggested that CRCs at 15.0 and 30.0 mg/kg effectively protected hepatic and renal functions in RM-induced AKI rats.

### 3.5 CRCs inhibited inflammatory response and oxidative stress in RM-induced AKI rats

**FIGURE 6:**
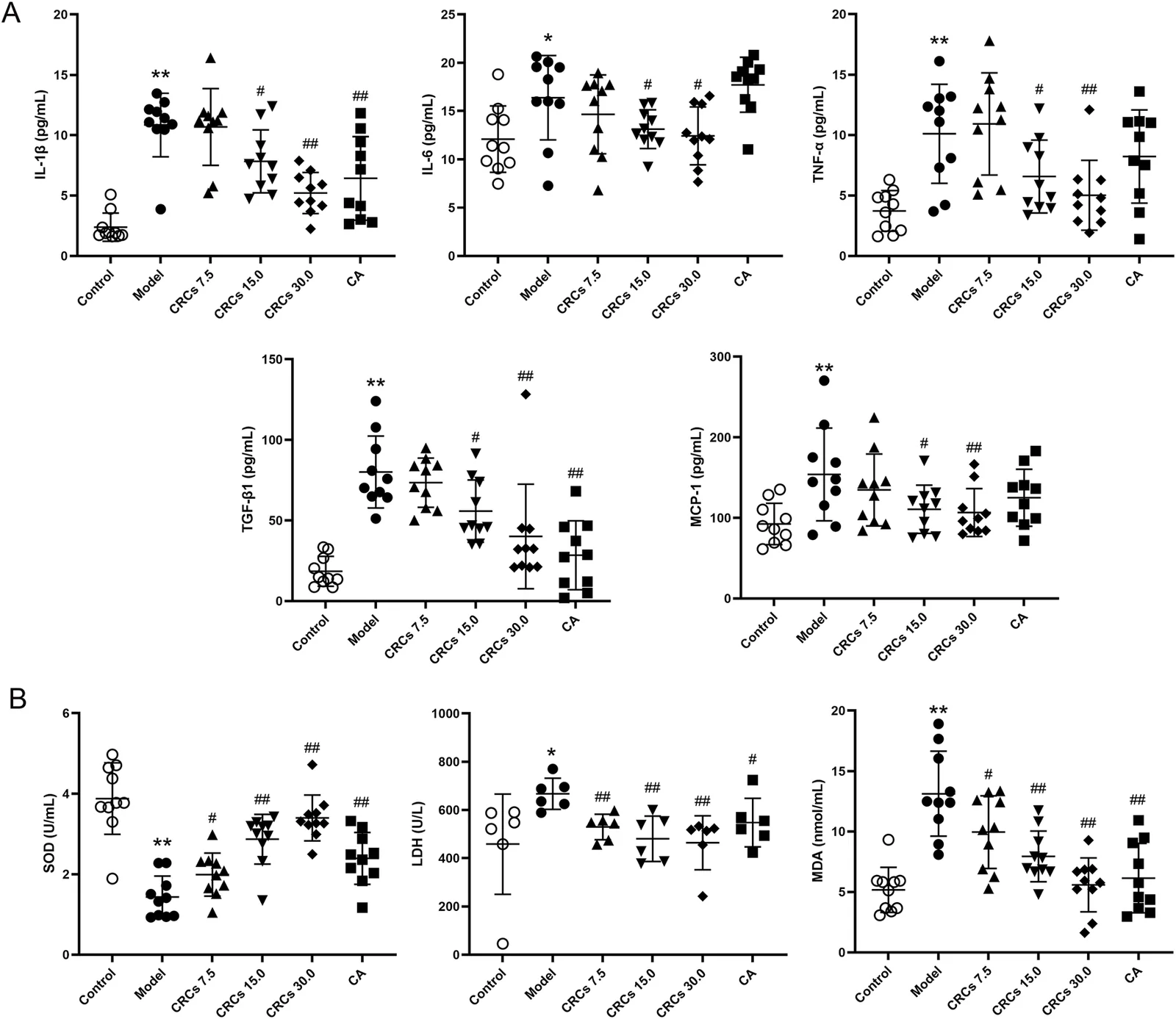
CRCs attenuated inflammation and oxidative stress in RM-induced AKI rats. **(A)** Serum inflammatory cytokines, including IL-1β, IL-6, TNF-α, TGF-β1, and MCP-1, measured by ELISA kits, n = 10. **(B)** Serum oxidative stress-related markers, including SOD, LDH, and MDA, determined by biochemical kits (SOD and MDA: n = 10; LDH: n = 6). Data are presented as mean ± SD. *p < 0.05, **p < 0.01 vs. control group; ^#^ *p* < 0.05, ^##^ *p* < 0.01 vs. model group.

Serum levels of inflammatory cytokines involving IL-1β, IL-6, TNF-α, TGF-β1, and MCP-1 were significantly higher in the model group than in the control group (Figure 6A). CRCs treatment at 15.0 and 30.0 mg/kg markedly reduced these cytokine levels. In addition, oxidative stress-related markers were altered in the model group, as evidenced by increased LDH activity and MDA content, along with reduced SOD activity (Figure 6B). CRCs administration significantly reversed these changes. These results indicated that CRCs exerted renoprotective effects in RM-induced AKI by attenuating inflammation and oxidative stress.

### 3.6 CRCs exerted renoprotective effects against RM-induced AKI through regulating the PLIN1/PPAR signaling pathway

**FIGURE 7:**
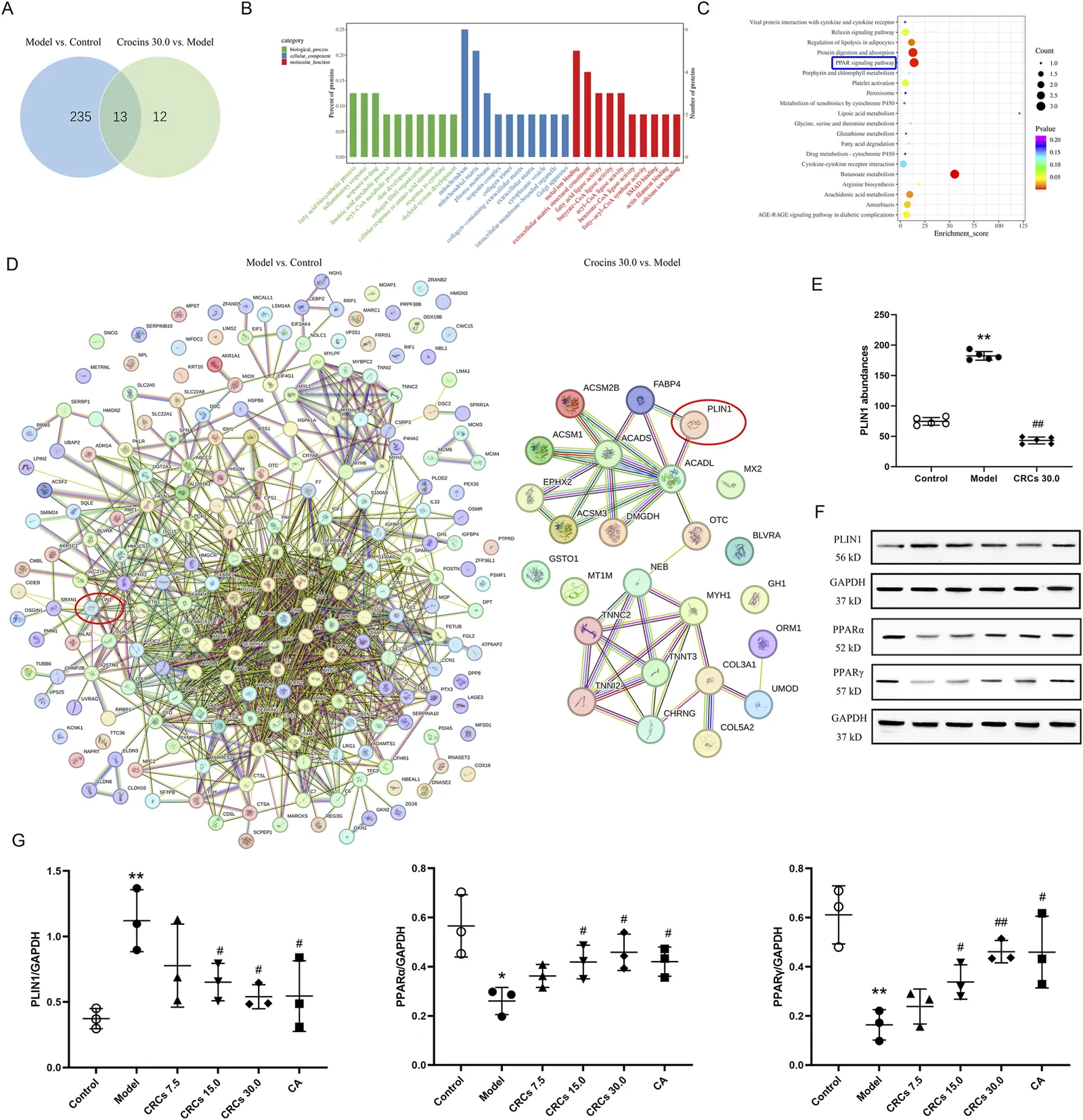
Quantitative proteomics and Western blotting validation demonstrated that CRCs protected against RM-induced AKI via the PLIN1/PPARs signaling pathway. **(A)** Venn diagram showing 13 overlapping DEPs between the comparisons of model vs. control and CRCs 30.0 mg/kg vs. model. **(B)** GO functional enrichment analysis of CRCs-regulated DEPs classified by BP, CC, and MF. **(C)** KEGG pathway enrichment analysis highlighting the top 20 signaling pathways. **(D)** PPI network analysis generated from the two comparisons. **(E)** Relative abundance of PLIN1 measured by TMT-based quantitative proteomics, n = 5. **(F)** Representative Western blotting bands of PLIN1, PPARα, and PPARγ. **(G)** Relative expression levels of PLIN1, PPARα, and PPARγ in renal tissue, n = 3. Data are expressed as mean ± SD. *p < 0.05, **p < 0.01 vs. control group; ^#^ *p* < 0.05, ^##^ *p* < 0.01 vs. model group.

Quantitative proteomics analysis identified 6,285 proteins across 15 samples. Compared with the control group, 197 proteins were significantly upregulated and 51 were downregulated in the model group (Figure 7A). In the CRCs-treated group, 25 DEPs were identified, including five upregulated and 59 downregulated proteins relative to the model group. There were 13 overlapping DEPs observed in both comparisons.

BP terms of GO enrichment analysis revealed that altered proteins in the CRCs group were mainly involved in fatty acid biosynthesis, inflammatory response, drug response, linoleic acid metabolism, and acyl-CoA metabolism. Enriched CC terms included mitochondria, mitochondrial matrix, and plasma membrane, while MF terms were linked to metal ion binding, extracellular matrix structural components, and fatty acid ligase activity (Figure 7B). KEGG pathway analysis showed that DEPs were associated with fatty acid degradation, arachidonic acid metabolism, PPAR signaling, glutathione metabolism, and xenobiotic metabolism by cytochrome P450 (Figure 7C).

To further clarify therapeutic targets of CRCs in RM-induced AKI, PPI network analysis was performed (Figure 7D), and perilipin 1 (PLIN1) emerged as a candidate protein (Figure 7E). KEGG analysis also linked PLIN1 to the PPARs signaling pathway. Western blotting confirmed that PLIN1 expression was significantly increased in the model group (1.47 ± 0.14) compared with the control group (1.00 ± 0.24). In contrast, PLIN1 expression was reduced in CRCs-treated groups (7.5 mg/kg, 1.04 ± 0.17; 15.0 mg/kg, 1.00 ± 0.12; 30.0 mg/kg, 0.82 ± 0.13), consistent with proteomics findings (Figures 7F,G).

As the PPARs signaling pathway was identified by both network analysis and proteomics, its role was further validated. Western blotting showed that PPARα and PPARγ expression levels were markedly downregulated in the model group compared with the control group (Figure 7G). Treatment with medium- and high-dose CRCs significantly restored PPARα and PPARγ expression. Taken together, these results demonstrated that CRCs protected against RM-induced AKI by regulating the PLIN1/PPAR signaling pathway.

**FIGURE 8:**
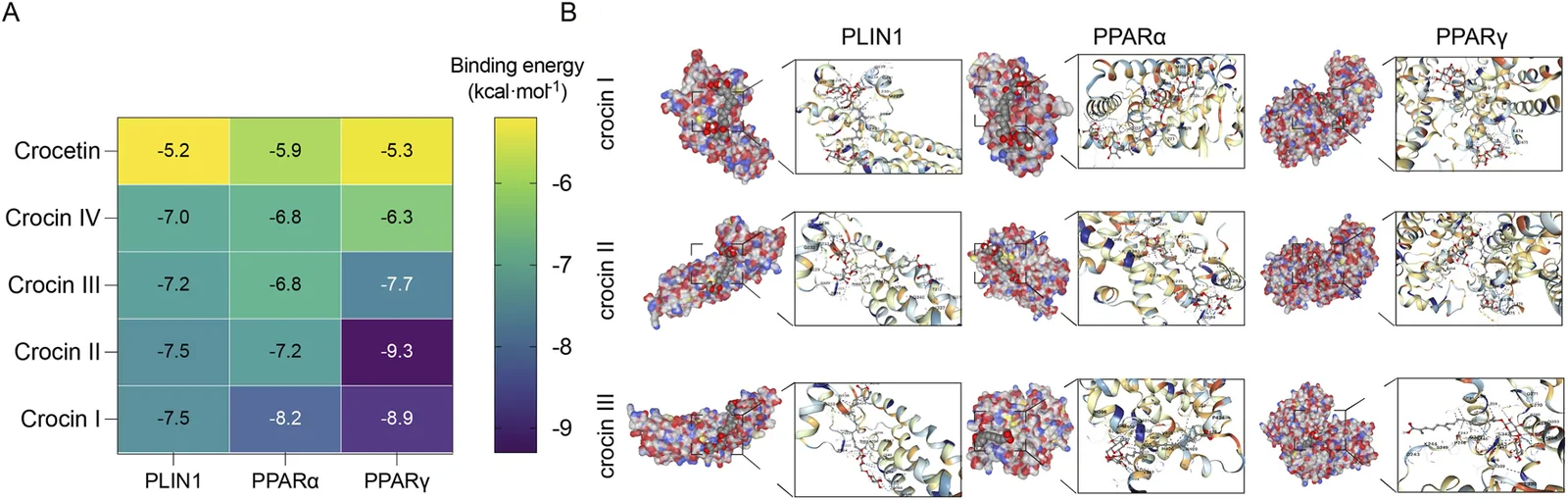
**(A)** Heat map showing molecular docking scores of the compounds and central targets. **(B)** Molecular docking 2D and 3D diagrams of the components and central targets as follows: PLIN1–crocin I/II (−7.5), PPARα–crocin I (−8.2), and PPARγ–crocin II (−9.3).

According to the result of network analysis, five chemical components of CRCs (crocin I, crocin II, crocin III, crocin IV, and crocetin) were selected for molecular docking with the three core targets: PLIN1, PPARα and PPARγ. Affinity < −7.0 kcal mol^−1^ is regarded as strong docking activity (Yan et al., 2026). The best docking activities were screened in PLIN1–crocin I/II (−7.5), PPARα–crocin I (−8.2), and PPARγ–crocin II (−9.3) (Figures 8A,B). Furthermore, crocin I and II showed good binding affinities with the corresponding targets, indicating their potential therapeutic effects in RM-induced AKI.

## 4 Discussion

Extensive studies have demonstrated that glycerol-induced myoglobinuric AKI is the most widely used animal model for investigating RM-induced AKI. Intramuscular injection of hypertonic glycerol triggers myolysis, hemolysis, and intravascular volume depletion. Serum CK serves as the most sensitive indicator of myocyte injury severity in RM. When muscle damage exceeds plasma protein binding capacity, circulating Mb reaches the glomeruli and is eventually excreted in the urine. The development of AKI in this context primarily involves three mechanisms: renal vasoconstriction, intraluminal cast formation, and direct cytotoxicity from heme proteins (Nath et al., 2022). Progressive renal injury exacerbates Mb accumulation, further impairing renal function. Consequently, serum Mb level correlates positively with renal function markers such as BUN and Scr, and serves as a valuable early predictor of RM and myoglobinuric AKI (Naso et al., 2026). Furthermore, approximately 25% of RM patients exhibit hepatic dysfunction due to proteases released from injured muscle, resulting in liver inflammation (Martinez et al., 2023). Severe RM cases may be accompanied by acute liver injury with elevated ALT and AST, posing a life-threatening condition (Yeh et al., 2022). In our study, CRCs significantly reduced serum levels of BUN, Scr, CK, Mb, ALT, and AST, as well as renal Mb content, while histopathological and immunohistochemical analyses confirmed that CRCs alleviated muscular and renal injuries in RM-induced AKI.

During renal filtration, Mb is endocytosed by tubular cells, where ferrous Mb is oxidized to ferric Mb, generating large amounts of reactive oxygen species (ROS). Ferric Mb is further converted to ferryl Mb via redox cycling, producing radical species that promote MDA synthesis, lipid peroxidation, and subsequent protein and DNA damages (Sil and Chakraborti, 2025). LDH, an oxidoreductase, participates in glycolysis, while SOD functions as a key antioxidant to eliminate ROS. Lipid peroxidation also generates F2 isoprostanes, potent vasoconstrictors that enhance intercellular adhesion molecule expression and pro-inflammatory cytokine production, leading to macrophage recruitment (Tu and Li, 2023). Our results showed that CRCs reduced LDH activity and MDA levels while increasing SOD activity, thereby mitigating oxidative stress.

RM-damaged muscle cells release immunostimulatory molecules that activate dendritic cells, T lymphocytes, and macrophages in renal tissue. These activated cells promote the production of pro-inflammatory cytokines, including IL-1β, IL-6, TGF-β, and TNF-α (Hebert et al., 2022). Mb-derived heme further exacerbates inflammatory responses in endothelial and tubular epithelial cells by upregulating chemokines such as IL-8 and activating neutrophil protein C (Tu and Li, 2023). The extent of macrophage infiltration and pro-inflammatory cytokine expression closely correlates with renal dysfunction and histological injury (Kassab et al., 2023). In our study, CRCs suppressed inflammatory responses by decreasing serum levels of IL-1β, IL-6, TNF-α, TGF-β1, and MCP-1.

PLIN1, a member of the PLIN family, is highly expressed on adipocyte surfaces and regulates lipid metabolism by protecting lipid droplets from lipolysis (Bombarda-Rocha et al., 2023). PLIN1 has been implicated in tumorigenesis, diabetes, atherosclerosis, and fatty liver disease through modulation of inflammation and lipid metabolism. Elevated PLIN1 levels in tissue and serum of patients with diabetic foot ulcers correlate positively with inflammatory cytokines such as IL-1β and TNF-α, and *in vitro* studies suggest that PLIN1 mediates inflammation via the NF-κB pathway (Wang et al., 2024). Dysregulation of PLIN1 through the PI3K/AKT pathway promotes tumor proliferation, invasion, and lipid metabolism reprogramming in glioma (Luo et al., 2025). PLIN1 also regulates macrophage inflammatory polarization by stabilizing lipid storage, influencing plaque stability and anti-inflammatory phenotypes (Cho et al., 2023). However, its role in RM-induced AKI had not been previously reported. Our proteomics results indicated that CRCs downregulate renal PLIN1, suggesting it as a potential therapeutic target in RM-induced AKI.

PPARs are nuclear hormone receptors comprising three major subtypes: PPARα, PPARβ/δ, and PPARγ (Christofides et al., 2021). PPARα is predominantly expressed in the liver, heart, brown adipose tissue, kidney, and intestine, whereas PPARβ/δ is highly expressed in the brain, stomach, and colon, and PPARγ is found in the heart, gut, adipose tissue, and immune cells (Wagner K. D. and Wagner, 2020). PPARα regulates fatty acid β-oxidation, lipid metabolism, and inflammatory responses, while PPARγ controls glucose and lipid metabolism, cell differentiation, and has anti-inflammatory and immunoregulatory roles. PPARβ/δ may function as a pro-angiogenic factor, though its precise role remains unclear (Wagner N. and Wagner, 2020). Previous studies reported that stevioside and umbelliferone protect against RM-induced AKI via PPARγ agonism (Kaur et al., 2021). In our study, Western blotting confirmed that CRCs modulated the PPARα and PPARγ signaling pathways, thereby exerting protective effects in RM-induced AKI. Molecular docking further revealed that the high binding affinity of the primary crocins (I–II) for PLIN1, PPARα and PPARγ. Our experimental data indicated that CRCs suppressed oxidative stress and inflammatory response by regulating the PLIN1/PPARs signaling pathway, thereby mitigating renal injury. This study may motivate clinical studies to assess the reproducibility and accuracy of these results in humans. If supported by clinical trials in the future, CRCs may offer a new prophylactic and therapeutic approach for RM-associated AKI.

Our study has several important limitations. First, our current data only support a correlative association. Functional rescue experiments including knockdown and overexpression should be performed to identify the causal relationship of the PLIN1/PPARs axis in RM-associated AKI. Second, the mechanistic investigation should be broadened and deepened such as revealing the roles of gut microbiota or downstream signalings regulated by PLIN1 in RM-associated AKI (Wang et al., 2026; Wu et al., 2026; Chang et al., 2025), thereby making our present study more complete and comprehensive. Third, since there are no authoritative therapeutic drugs currently available for the treatment of RM-induced AKI, catechins was employed as the positive control drug in the present study based on the reported literature. The positive drug may be not suitable for further exploring the accurate mechanisms. Fourth, the pharmacokinetic profile of CRCs and their active metabolites in blood and renal tissue was not monitored, leaving the optimal therapeutic regimen undefined. Moreover, research shows that crocin is not readily absorbed in the intestinal tract and it is then hydrolyzed to crocetin, absorbed into the circulation. Therefore, due to the low oral bioavailability, CRCs are more appropriate for adjunctive use in mild-to-moderate RM or in the convalescent phase. Despite these limitations, our findings provide the first integrative evidence for the renoprotective potential of CRCs in RM-induced AKI and offer a solid foundation for future mechanistic and translational studies.

## 5 Conclusion

**FIGURE 9:**
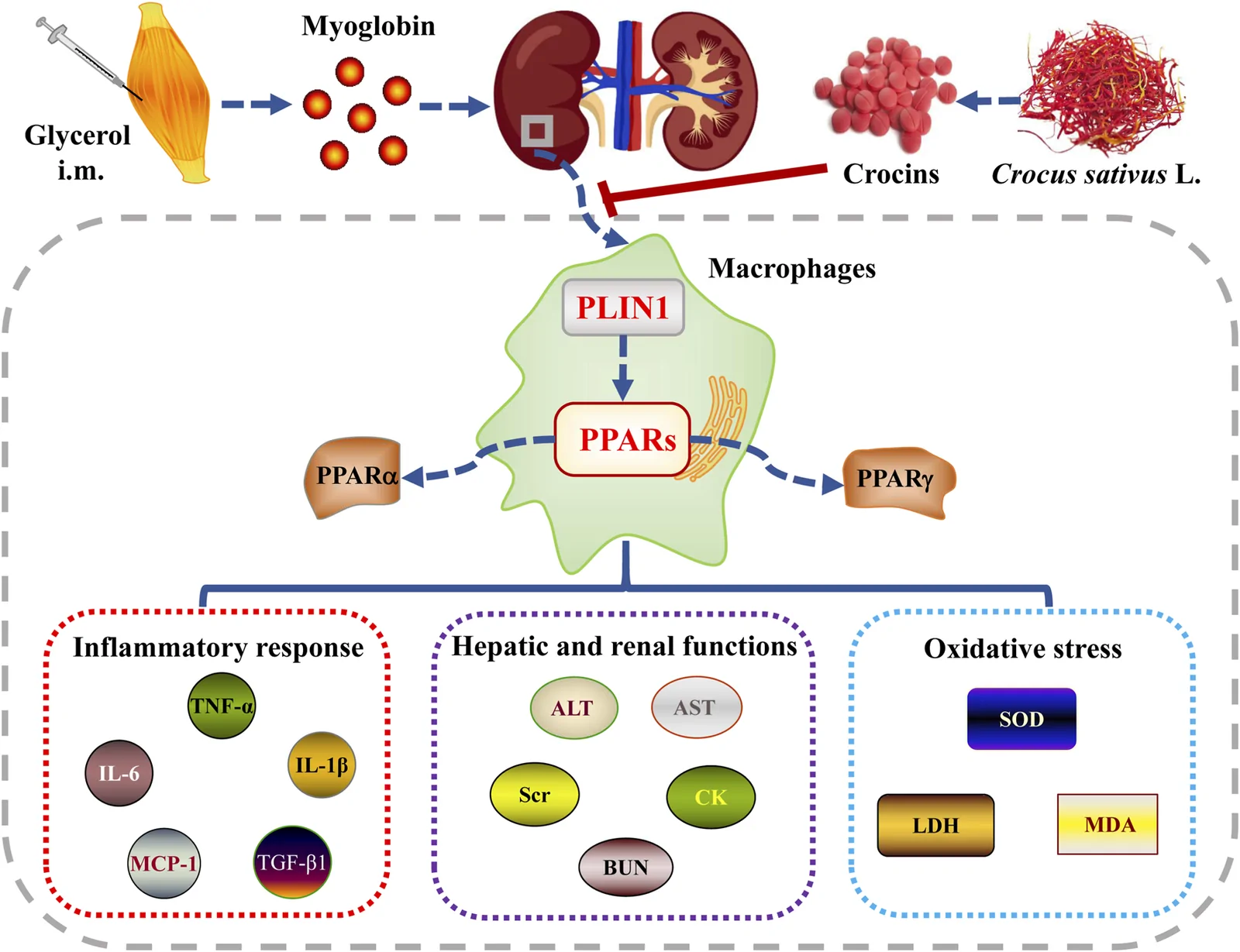
Schematic illustration of the protective effects of CRCs against hypertonic glycerol-induced myoglobinuric AKI via modulation of the PLIN1/PPARs signaling pathway.

In summary, network analysis identified potential molecular targets of CRCs in RM treatment. Animal experiments demonstrated that CRCs inhibited inflammation and oxidative stress, preserved hepatic and renal functions, and alleviated muscle and renal injuries induced by intramuscular glycerol injection. Quantitative proteomics and molecular docking further highlighted PLIN1 as a key biomarker in the therapeutic mechanism of CRCs (Figure 9). Collectively, our findings provide the first direct evidence that CRCs protect against RM-induced AKI through modulating the PLIN1/PPARs signaling pathway and offer experimental support for potential clinical applications in patients with RM.

## Data Availability

The original contributions presented in the study are included in the article/supplementary material, further inquiries can be directed to the corresponding authors.
